# Cerebrospinal fluid ctDNA testing shows an advantage over plasma ctDNA testing in advanced non-small cell lung cancer patients with brain metastases

**DOI:** 10.3389/fonc.2023.1322635

**Published:** 2024-01-10

**Authors:** Xiaocui Liu, Fengjun Mei, Mei Fang, Yaqiong Jia, Yazhu Zhou, Chenxi Li, Panpan Tian, Chufan Lu, Guangrui Li

**Affiliations:** ^1^ Department of Neurology, The Fourth Hospital of Hebei Medical University, Shijiazhuang, Hebei, China; ^2^ Department of Neurology, North China University of Science and Technology Affiliated Hospital, Tangshan, Hebei, China; ^3^ Department of Reproductive Medicine, The Fourth Hospital of Hebei Medical University, Shijiazhuang, Hebei, China; ^4^ Department of Neurology, The First Hospital of Hebei Medical University, Shijiazhuang, Hebei, China; ^5^ Department of Infectious Diseases, The Third Hospital of Hebei Medical University, Shijiazhuang, Hebei, China

**Keywords:** non-small cell lung cancer (NSCLC), leptomeningeal metastases (LM), brain parenchyma metastases (BPM), cerebrospinal fluid circulating tumour DNA (ctDNA), next generation sequencing

## Abstract

**Background:**

Brain metastases (BM), including brain parenchyma metastases (BPM) and leptomeningeal metastases (LM), are devastating metastatic complications in advanced cancer patients. Next-generation sequencing (NGS) is emerging as a new promising tool for profiling cancer mutation, which could facilitate the diagnosis of cancer. This retrospective study aimed to investigate the molecular genetic characteristics of non-small cell lung cancer (NSCLC) patients with BPM and LM using NGS.

**Methods:**

Cerebrospinal fluid (CSF) samples and paired plasma samples were collected from 37 patients of NSCLC-BM. We profiled genetic mutation characteristics using NGS from NSCLC-BM by comparing CSF circulating tumour DNA (ctDNA) with plasma ctDNA and primary tumour tissues.

**Results:**

Among the 37 patients with NSCLC-BM, 28 patients had LM with or without BPM, while 9 patients only had BPM. Driver and drug-resistant mutations in primary tumours with LM included: *EGFR* L858R (10, 35.7%), *EGFR* 19del (6, 21.4%), *EGFR* L858R+*MET* (1, 3.6%), *EGFR* L858R+S768I (1, 3.6%), *ALK* (2, 7.1%), *ROS1* (1, 3.6%), negative (5, 17.9%), and unknown (2, 7.1%). In patients with NSCLC-LM, the detection rate and abundance of ctDNA in the CSF were significantly higher than those in paired plasma. The main driver mutations of NSCLC-LM remained highly consistent with those of the primary tumours, along with other unique mutations. Circulating tumour DNA was negative in the CSF samples of BPM patients. Patients with BMP had a higher ratio of *EGFR* 19del than L858R mutation (55.6% vs 11.1.%), whereas NSCLC patients with LM had a higher ratio of *EGFR* L858R than 19del mutation (50.0% vs 25.0%). Most patients with positive plasma ctDNA results were male (*p* = 0.058) and in an unstable state (*p* = 0.003).

**Conclusion:**

Our study indicated that the CSF ctDNA detected by NGS may reflect the molecular characteristics and heterogeneity of NSCLC-LM. Timely screening of patients with NSCLC for CSF ctDNA, especially for patients with positive plasma ctDNA, may facilitate the early detection of LM. Furthermore, patients with the *EGFR* 19del may have a higher risk of developing BPM.

## Background

Brain metastases (BM), including brain parenchyma metastases (BPM) and leptomeningeal metastases (LM), are devastating complications in advanced cancer patients. Lung cancer is the leading cause of brain metastases (BM). Patients with advanced non-small cell lung cancer (NSCLC) are often present with metastatic disease, including 64.5% with BPM and 35.5% with LM (1). Epidermal growth factor receptor (*EGFR*) mutations occur in 9.4% of NSCLC patients (2). The most common occurrence site of BPM is the cerebral hemisphere, followed by the cerebellum and brainstem (3). LM is defined as cancer cells disseminating to both the leptomeninges (pia and arachnoid) and cerebrospinal fluid (CSF) compartment and often results in significant neurological morbidity. The diagnosis of LM usually relied on magnetic resonance imaging (MRI) with gadolinium enhancement and CSF cytology. Notably, CSF cytology is the gold standard for diagnosing LM. CSF cytological evaluation has high specificity but only moderate sensitivity with a positive rate of the first test was 50% (4). MRI has limited sensitivity, and the sensitivity of this diagnostic modality has not yet been firmly established. Existing treatment options for BM primarily include radiation therapy, systemic chemotherapy, targeted therapy, intrathecal chemotherapy, and immune checkpoint inhibitors, among which targeted therapy can improve the prognosis and quality of life in NSCLC-BM patients with sensitive mutations. However, the prognosis of patients with NSCLC-BM remains poor, with a median overall survival (OS) of 36.3 months for patients with BPM and 26.4 months for patients with LM (1).

Genomic characterisation of NSCLC-BM is crucial for its precise diagnosis and treatments. However, obtaining brain tissues is difficult, which hinders our understanding of BM’s genetic status. With the development of liquid biopsy, plasma and CSF circulating tumours (ctDNA) detected by next-generation sequencing (NGS) play an increasingly important role in guiding the management of NSCLC-LM. For metastatic and/or recurrent disease, the advantages of liquid biopsy over tissue biopsy are non-invasive, repeatable, and the possibility to obtain a full overview of the genetic makeup of the disease, overcoming both spatial and temporal heterogeneity (5). However, plasma ctDNA levels do not fully reflect genetic mutations in patients with BM. A previous study indicated that CSF-derived ctDNA detected by NGS shows higher sensitivity than plasma ctDNA and better reflects the genetic profile of patients with LM (6). In this retrospective study, we aimed to investigate the ctDNA molecular genetic characteristics of patients with non-small cell lung cancer patients with BPM and LM by NGS.

## Patients and methods

### Patients

**Figure 1:**
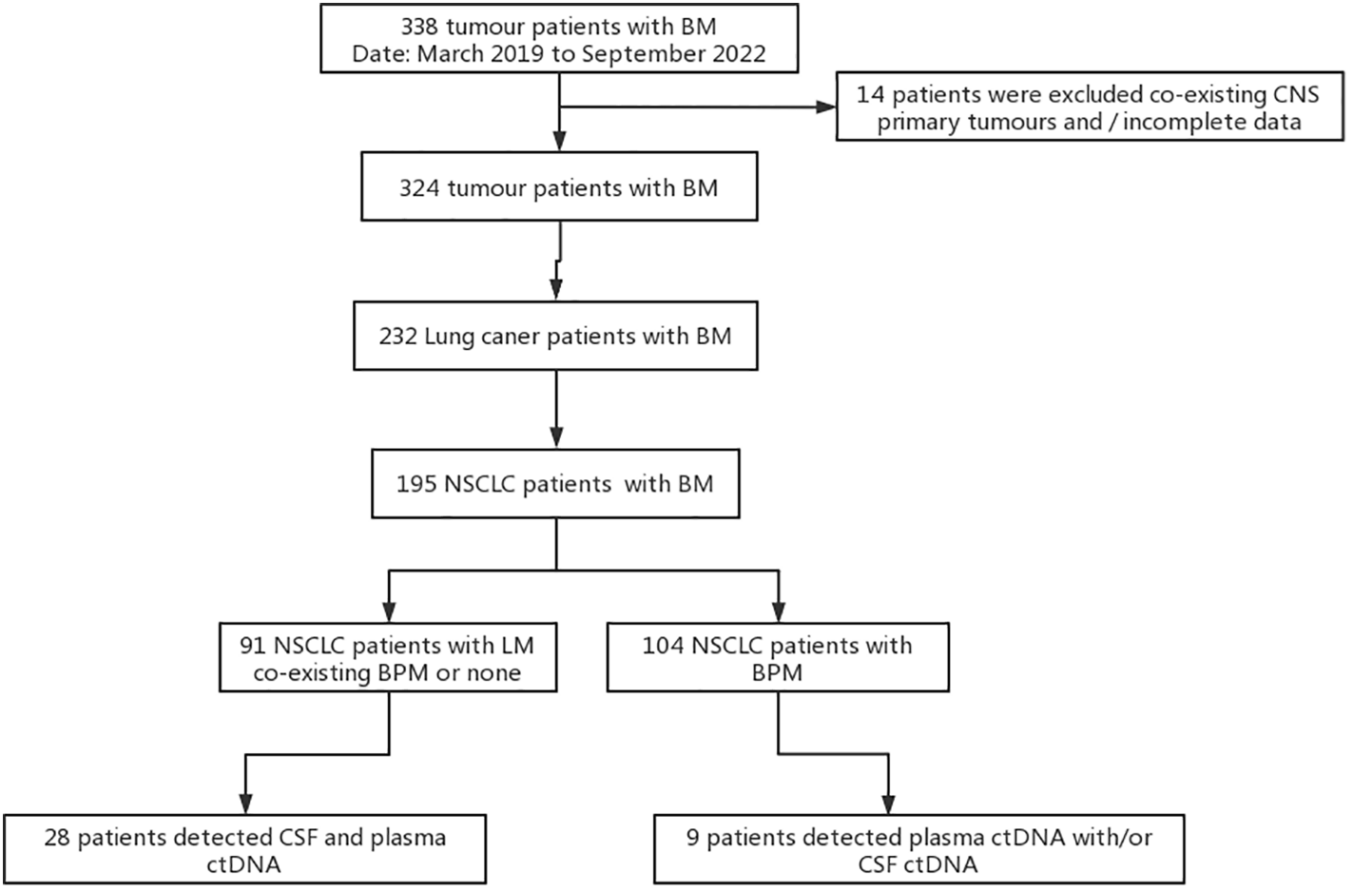
Study flowchart. BM, brain metastases; BPM, brain parenchyma metastases; LM, leptomeningeal metastases; CSF, cerebrospinal fluid circulating tumour; ctDNA, circulating tumour DNA; NSCLC, non-small cell lung cancer.

In total, 28 patients with NSCLC-LM and 9 patients with NSCLC-BPM were enrolled between March 2019 and September 2022 at the Neurology Department of the Fourth Hospital of Hebei Medical University. Inclusion criteria: (1) the primary tumour was confirmed as NSCLC by pathology or cytology; (2) LM was confirmed according to the 2017-ESMO guidelines, including 1) a clear history of tumour, 2) new neurological signs, 3) typical imaging manifestations, 4) cancer cells were found in CSF cytology; (3) the diagnostic criteria for BPM were based on a positive result on brain MRI; and (4) NGS testing of samples, including CSF, plasma, and/or tissues. Exclusion criteria were as follows: (1) CSF and plasma ctDNA were not paired and (2) co-existing primary tumour of the brain or spinal cord (**Figure 1**).

The research was conducted according to the principles set out in the Declaration of Helsinki 1964. All subsequent revisions and informed consent were obtained and the study was approved by the Institutional Review Board and Ethics Committee of the Fourth Hospital of Hebei Medical University (approval 2022KS004).

### Data collection

Data on the initial diagnosis of LM or BPM were collected in the medical records of enrolled patients, including sex, age, Eastern Cooperative Oncology Group Performance Status (ECOG PS) score, extracranial disease status, BPM status, driver and drug-resistant mutations of primary tumours, NGS results, CSF parameters, imaging examination results, and treatment history (**Table 1**). Extracranial disease progression was defined using the Response Evaluation Criteria in Solid Tumours (RECIST) version 1.1 (7). BPM progression was defined using the Response Assessment in Neuro-oncology Brain Metastases (RANO-BM).

**Table 1:** Clinical information of non-small cell lung cancer with leptomeningeal metastases.

NO.	Age	Sex	Driver mutation of primary tumour	PS	Metastactic site	Extracranial disease status	BPM status	Therapy before CSF collection	TKIs after LM
1	55	F	(-)	3	BPM/LM	without	PD	None	None
2	47	F	L858R	2	LM	without	without	None	osimertinib
3	57	F	L858R	2	BPM/LM/bone/Lymph node	PD	SD	gefitinib	osimertinib
4	52	M	ROS1	2	LM/Bone/Lymph node	SD	without	crizotinib	Lorlatinib
5	62	M	L858R	1	BPM/LM	without	SD	None	almonertinib/osimertinib
6	54	M	(-)	2	LM/Bone	SD	without	almonertinib	Sevatinib/furmonertinib
7	57	M	19del	1	BPM/LM/Pleural/Lymph node	SD	PD	gefitinib	osimertinib
8	59	M	(-)	1	BPM/LM	without	SD	None	None
9	64	F	L858R	3	BPM/LM/Lymph node	SD	PD	icotinib	osimertinib
10	65	F	L858R	3	LM/Bone	SD	without	Icotinib/osimertinib	osimertinib
11	62	M	(-)	2	BPM/LM	PD	PD	gefitinib	osimertinib
12	50	F	(-)	3	BPM/LM/Bone/spinalis/Lymph node	SD	SD	osimertinib	Osimertinib/crizotinib
13	74	F	L858R	1	BPM/LM/Bone	PD	PD	gefitinib	osimertinib
14	70	F	ALK	1	BPM/LM/Bone/abdomen/Lymph node	PD	PD	crizotinib	bugatinib
15	53	F	ALK	1	BPM/LM	without	PD	Crizotinib/ceritinib	alectinib
16	66	F	19del	1	BPM/LM/pulmonary	SD	PD	gefitinib	osimertinib
17	70	M	19del	1	BPM/LM/Bone/Pleural/peritoneum	PD	SD	Icotinib/gefitinib/almonertinib	almonertinib
18	39	F	19del	0	BPM/LM/Bone/Lymph node	PD	PD	Gefitinib/erlotinib	erlotinib
19	48	M	L858R	2	LM/bone	PD	without	None	osimertinib
20	67	M	L858R	1	LM	without	without	gefitinib	Almonertinib/furmonertinib
21	71	M	L858R	2	BPM/LM/Bone/adrenal	PD	PD	osimertinib	Almonertinib
22	72	M	19del	4	LM/Lymph node	SD	without	None	erlotinib
23	54	F	L858R	2	BPM/LM/Bone	PD	SD	icotinib	osimertinib
24	50	M	19del	3	BPM/LM/Bone/Pleural/adrenal	PD	PD	gefitinib	Erlotinib/Almonertinib/osimertinib
25	34	F	L858R/MET	2	LM/Bone/pulmonary	SD	without	Icotinib/osimertinib	osimertinib/dacomitinib/crizotinib
26	52	M	unknown	2	LM/Bone	PD	without	Icotinib/osimertinib	afatinib
27	56	F	unknown	3	LM/Bone/spinalis	SD	without	icotinib	None
28	51	M	L858R+ S768I	1	BPM/LM/Lymph node	SD	PD	icotinib	Osimertinib/afatinib

BPM, brain parenchyma metastases; LM, leptomeningeal metastases; CSF, cerebrospinal fluid circulating tumour; TKIs, tyrosine kinase inhibitors: Disease stability assessment: CR, complete response; PR, partial response; SD, stable disease; PD, progress disease.

### Circulating tumour DNA extraction and library construction

First, 5ml of whole blood was collected by EDTA blood collection tubes and then centrifuged within 1 hour of collection at 1,800×g for 10 minutes at 4≥°C or room temperature to remove the blood cells. The supernatant containing the plasma was removed with special care taken so as to not disturb the buffy coat. This was then centrifuged at 16,000× g for 10 minutes to remove any remaining cells. ctDNA was extracted from 2ml plasma, by digestion in 100μl proteinase K buffer for 10min at 37°C followed by purification with the NucleoSpin Plasma XS kit with modified protocols. The purified ctDNA was quantified by a Picogreen fluorescence assay using the provided lambda DNA standards (Invitrogen). Then, library construction with the KAPA Hyper DNA Library Prep Kit, containing mixes for end repair, dA addition and ligation, was performed in 96-well plates (Eppendorf). Dual-indexed sequencing libraries were PCR amplified for 4-7 cycles.

### Hybrid selection and ultra-deep NGS of ctDNA

The 5’-biotinylated probe solution was provided as capture probes, the baits targeted 416 cancer-related genes. Furthermore, 1μg of each ctDNA-fragment sequencing library was mixed with 5μg of human Cot-1 DNA, 5μg of salmon sperm DNA, and 1 unit adaptor-specific blocker DNA in hybridisation buffer, heated for 10 minutes at 95°C, and held for 5 minutes at 65°C in the thermocycler. Within 5 minutes, the capture probes were added to the mixture, and the solution hybridisation was performed for 16-18 hours at 65°C. After hybridisation was complete, the captured targets were selected by pulling down the biotinylated probe/target hybrids using streptavidin-coated magnetic beads, and the off-target library was removed by washing with wash buffer. The PCR master mix was added to directly amplify (6-8 cycles) the captured library from the washed beads. After amplification, the samples were purified by AMPure XP beads, quantified by qPCR (Kapa) and sized on a Bioanalyzer 2100 (Agilent). Libraries were normalised to 2.5nM and pooled. Deep Sequencing was performed on Illumina HiSeq 4000 using PE75 V1 Kit. Cluster generation and sequencing was performed according to the manufacturer’s protocol.

### Sequence alignment and processing

Base calling was performed using bcl2fastq v2.16.0.10 (Illumina, Inc.) to generate sequence reads in FASTQ format (Illumina 1.8+ encoding). Quality control (QC) was applied with Trimmomatic. High-quality reads were mapped to the human genome (hg19, GRCh37 Genome Reference Consortium Human Reference 37) using modified BWA aligner 0.7.12 with BWA-MEM algorithm and default parameters to create SAM files. Picard 1.119 (http://picard.sourceforge.net/) was used to convert SAM files to compressed BAM files which were then sorted according to chromosome coordinates. The Genome Analysis Toolkit (GATK, version 3.4-0) was modified and used to locally realign the BAM files at intervals with indel mismatches and recalibrate base quality scores of reads in the BAM files.

### SNVs/Indels/CNVs detections

Single nucleotide variants (SNVs) and short insertions/deletions (indels) were identified using VarScan2 2.3.9 with the minimum variant allele frequency threshold set at 0.01 and the p-value threshold for calling variants set at 0.05 to generate Variant Call Format (VCF) files. All SNVs/indels were annotated with ANNOVAR, and each SNV/indel was manually checked with the Integrative Genomics Viewer (IGV). Copy number variations (CNVs) were identified using ADTEx 1.0.4. In total, 425 cancer-related genes are listed in **Supplementary Table 1**.

### Statistical analysis

Due to the relatively small sample size, only descriptive statistics were used. Data are presented as numbers and percentages.

## Results

### Baseline characteristics of NSCLC-BM patients

**Figure 2:**
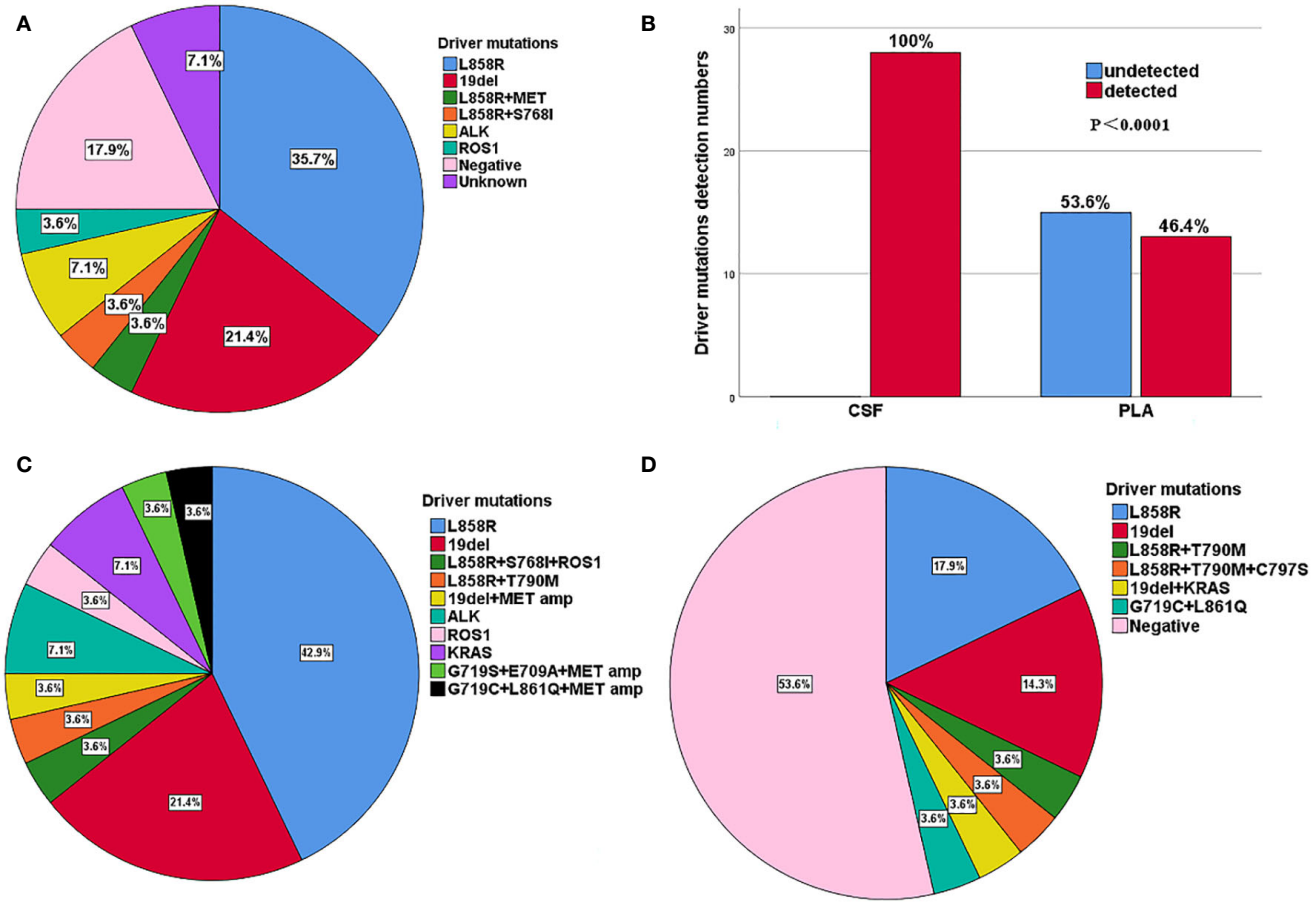
**(A)** Distribution of driver mutations of primary tumour in 28 patients with LM. **(B)** Comparison of driver gene mutations in CSF and plasma of 28 patients with LM. **(C)** Distribution of mainly gene mutations in CSF for 28 patients with LM. **(D)** Distribution of mainly gene mutations in plasma for 28 patients with LM.

Among the 37 patients with NSCLC, 28 had LM with or without BPM, while 9 patients had only BPM. Of the 28 patients with LM, 14 (50.0%) were female, and the median age was 57 years (range: 34–74 years). LM was diagnosed either at baseline (n = 3, 10.7%) or during the treatment course (n = 25, 89.3%), with a median interval of 19.0 (1.0–70.0) months. The main driver mutations of primary tumours with LM were determined by NGS, including *EGFR* L858R (10, 35.7%), *EGFR* 19del (6, 21.4%), *EGFR* L858R + *MET* amplification (1, 3.6%), *EGFR* L858R+S768I (1, 3.6%), *ALK* (2, 7.1%), *ROS1* (1, 3.6%), negative (5, 17.9%), and unknown (2, 7.1%) (**Figure 2A**). The ECOG PS scores at the time diagnosis of LM were as follows: 0 (1, 3.6%), 1 (10, 35.7%), 2 (10, 35.7%), 3 (6, 21.4%), 4 (1, 3.6%). Of the 28 patients, 18 (64.3%) had BPM at the initial diagnosis of LM. In total, 22 patients (78.6%) had an extracranial disease. The BPM status was as follows: PD (12, 42.9%), SD (6, 21.4%), and no BPM (10, 35.7%). Extracranial disease status was as follows: PD (11, 39.3%), SD (11, 39.3%), and no extracranial disease (n = 6, 21.4%). Most patients (23 of 28, 82.1%) with driver mutations had a history of targeted therapies and were switched to a different targeted drug upon the disease progression (**Table 1**).

### CSF ctDNA showed a higher sensitivity than plasma ctDNA in NSCLC-LM patients

**Figure 3:**
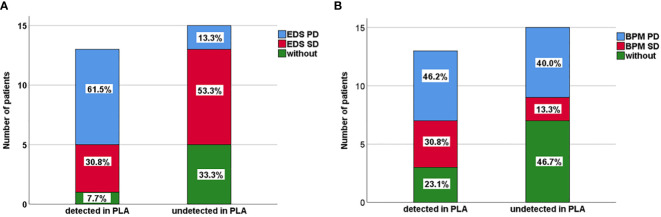
**(A)** Extracranial disease status (EDS) of 13 patients positive with plasma ctDNA and 15 patients negative with plasma ctDNA in NSCLC-LM patients. **(B)** Brain parenchyma metastases (BPM) status of 13 patients positive with plasma ctDNA and 15 patients negative with plasma ctDNA in NSCLC-LM patients.

**Figure 4:**
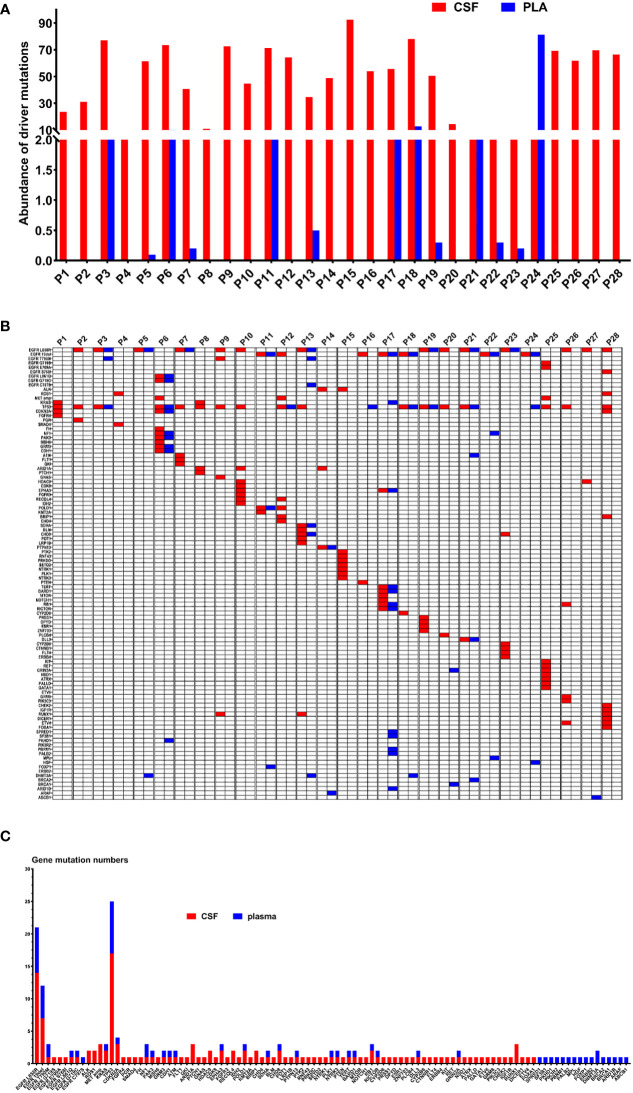
**(A)** The abundance of ctDNA in the CSF and plasma of 28 patients with LM. **(B)** Genetic profiles of 28 LM patients in the CSF and the paired plasma. **(C)** The gene mutation numbers of 28 LM patients in the CSF and the paired plasma. Red: CSF; Blue: plasma.

The results indicated that CSF ctDNA demonstrated higher sensitivity compared to plasma ctDNA in detecting mutations in LM. All CSF ctDNA were positive, while 13 of 28 (46.4%) plasma ctDNA were positive (**Figure 2B**). The main driver and drug-resistant mutations detected in CSF include *EGFR* L858R (12, 42.9%), *EGFR* 19del (6, 21.4%), *EGFR* L858R+S768I+*ROS1* (1, 3.6%), *EGFR* L858R+T790M (1, 3.6%), *EGFR* 19del+*MET* amplification (1, 3.6%), *ALK* (2, 7.1%), *ROS1* (1, 3.6%), *KRAS* (2, 7.1%), *EGFR* G719S+E709A+*MET* amplification (1, 3.6%), and *EGFR* G719C+L861Q+*MET* amplification (1, 3.6%) (**Table 2**, **Figure 2C**). Mutations detected in plasma include *EGFR* L858R (5, 17.9%), *EGFR* 19del (4, 14.3%), *EGFR* L858R+T790M (1, 3.6%), *EGFR* L858R+T790M+C797S (1, 3.6%), *EGFR* 19del+*KRAS* (1, 3.6%), *EGFR* G719C+L861Q (1, 3.6%), and negative (15, 53.6%) (**Table 2**, **Figure 2D**). The detection rate and types of ctDNA in the CSF were higher than those in paired plasma samples. The consistency of main mutations between the CSF and the paired plasma was 32.1% (9/28). Most of patients with positive driver mutations in plasma ctDNA were male (M:F = 9:4), and their extracranial disease state was more likely to be at a progressive stage (8/13, 61.5%) (**Figure 3A**, **Supplementary Figure 1**). Conversely, the majority of patients without driver mutations in plasma were female (M:F = 5:10), whose extracranial disease state was more likely at a stable stage/without extracranial disease (13/15, 86.7%) (**Figure 3A**, **Supplementary Figure 1**). Moreover, 76.9% (10/13) of NSCLC-LM patients, who were positive for plasma ctDNA, had BPM, compared with 53.3% (8/15) of patients without plasma ctDNA (**Table 1**, **Figure 3B**). The abundance of CSF ctDNA was significantly higher than that of plasma ctDNA, except in one patient (P24) who had a higher abundance of driver mutations in the plasma sample than CSF sample and his extracranial disease was in progression with new bone metastases at the time of the initial diagnosis of LM (**Table 1**, **Figure 4A**).

**Table 2:** The results of gene mutations of non-small cell lung cancer with leptomeningeal metastases.

NO.	Driver mutations of primary tumours	Interval time (month)	Tumour tissues	Abundance(tissues) (%)	CSF ctDNA	Abundance (CSF) (%)	Plasma ctDNA	Abundance (plasma) (%)
1	(-)	1			KRAS	23.7	(-)	
2	L858R	0			L858R	31.1	(-)	
3	L858R	17			L858R	77.1	L858R/T790M	5.6
4	ROS1	32			ROS1	10.0	(-)	
5	L858R	54			L858R	61.5	L858R	0.1
6	(-)	15			G719C/L861Q/MET	73.7/67.3/3.35	G719C/L861Q	10.1/11.3
7	19del	28			L858R	40.7	L858R	0.2
8	(-)	19	KRAS	10.13	KRAS	10.9	(-)	
9	L858R	38			L858R/T790M	72.6/9.7	(-)	
10	L858R	45			L858R	44.8	(-)	
11	(-)	19			19del	71.4	19del	8.8
12	(-)	15			19del/MET	64.3/2.09	(-)	
13	L858R	17			L858R	34.6	L858R/T790M/C797S	0.5/0.28/0.1
14	ALK	14			ALK	48.9	(-)	
15	ALK	23	ALK	54.82	ALK	92.6	(-)	
16	19del	6			19del	54.0	(-)	
17	19del	22			19del	55.7	19del/KRAS	8.7/1.46
18	19del	20			19del	78.1	19del	12.9
19	L858R	0			L858R	50.5	L858R	0.3
20	L858R	8			L858R	14.5	(-)	
21	L858R	0	L858R	22.7	L858R	8.4	L858R	4.4
22	19del	35			19del	7.4	19del	0.3
23	L858R	3			L858R	4.6	L858R	0.2
24	19del	12	19del	56.7	19del	8.0	19del	81.4
25	L858R/MET	67	G719S/E709A	20.85/21.33	G719S/E709A/MET	69.2/69.6/3.14	(-)	
26	unknown	70			L858R	61.8	(-)	
27	unknown	45			L858R	69.6	(-)	
28	L858R+E S768I	17			L858R+S768I+ROS1	66.5/69.5/6.1	(-)	

NSCLC, non-small cell lung cancer; CSF, cerebrospinal fluid circulating tumour; ctDNA, circulating tumour DNA.

### The consistency of CSF and plasma ctDNA with driver mutations of primary tumours for NSCLC-LM

The main driver mutations observed in NSCLC-LM demonstrated high consistency with those found in primary tumours. Mutations in the CSF samples showed greater concordance with the primary tumour mutations. Among the 21 patients with positive driver mutations in their primary tumours, 19 (90.5%) patients showed driver mutations in the CSF. In two patients, who did not show driver mutations in primary tumours, *EGFR* mutations were detected in the CSF, including 19del mutation in patient 7 (which later changed to L858R mutation after 28 months), and L858R+MET amplification in patient 25 (which later changed to G719S+E709A+MET amplification after 67 months). Of these 21 patients, 10 (47.6%) of them were driver mutation positive in plasma samples (**Tables 1**, **2**).

Five of the 28 patients with NSCLC-LM underwent both tumour tissue biopsy and paired CSF NGS, with a high consistency between the two sample types. While driver mutations had been detected in the CSF sample of the five patients, who were driver mutation negative in the primary tumour, only two of them had driver mutations detected in the paired plasma sample. One drug-resistant mutation (*MET*) was also detected in the CSF but not in the tumour tissues (**Table 2**).

### Genomic profiles of NSCLC-LM

**Figure 4B** displays genomic profiles of the 28 NSCLC-LM cases in both the CSF and paired plasma. The primary driver mutations were observed in the *EGFR* gene. The rare mutations were partially different between CSF and plasma samples. The average number of mutations identified in the CSF was 4.9 (range, 0–12). The most frequently mutated genes were *TP53* (17, 60.7%), *EGFR* L858R (14, 42.9%), *EGFR* 19del (7, 21.4%), *ARID1A* (3, 10.7%), *CDKN2A* (3, 10.7%), *MET* (3, 10.7%), *RUNX1* (3, 10.7%), *ALK* (2, 7.1%), *ROS1* (2, 7.1%), *CHD8* (2, 7.1%), *HDAC9* (2, 7.1%), *KRAS* (2, 7.1%), *POLD1* (2, 7.1%), *EPHA3* (2, 7.1%), *RECQL4* (2, 7.1%), *BRIP1* (2, 7.1%), and *RB1* (2, 7.1%) (**Figures 4B, C**). The average number of different mutations identified in the plasma was 2.3 (range 0–12). The frequently altered genes were *TP53* (8, 28.6%), *EGFR* L858R (7, 25.0%), *EGFR* 19del (5, 17.9%), *EGFR* T790M (2, 7.1%), *DNMT3A* (2, 7.1%), and *NF1* (2, 7.1%) (**Figures 4B, C**). The *EGFR* T790M mutation was detected in two plasma samples (P3 and P13) and one CSF sample (P9). All the patients were treated with first-generation *EGFR*-tyrosine kinase inhibitors (TKIs) before testing. *MET* amplification was detected in three CSF samples (P6, P12, and P25), treated with third-generation *EGFR*-TKIs. Additionally, some rare concomitant mutations, including *EGFR* G719C+L861Q+*MET* amplification (1, 3.6%), *EGFR* G719S+E709A+*MET* amplification (1, 3.6%), and *EGFR* L858R+S768I + *ROS1* (1, 3.6%), were also detected in the CSF samples. Furthermore, *EGFR* L858R+C797S+T790M (1, 3.6%) was detected in the plasma samples (**Table 2**, **Figure 4B**).

### CSF and plasma ctDNA for NSCLC-BPM

Among the nine patients with BPM, driver mutations of primary tumours were determined by NGS, including *EGFR* mutations (5, 55.6%), which include 19del (4, 44.4%), L858R+A871E + *MET* (1, 11.1%), *ALK* (1, 11.1%), *ROS1* (1, 11.1%), *KRAS* (1, 11.1%), and unknown (1, 11.1%). CSF ctDNA tests were performed in seven patients, all of whom tested negative for ctDNA. For the five patients initially diagnosed with BPM, both tissues and paired plasma were tested in four patients, with two of them showing consistency (2/4, 50.0%). In four patients diagnosed with BPM during the course of the disease, three patients had explicit genetic mutations, with one patient’s driver mutations being consistent in plasma ctDNA and primary tumour tissues (1/3, 33.3%), along with T790M resistant mutation only detected in the plasma. It is worth noting that while plasma ctDNA may be useful in BPM as a complementary assay for tissue analysis, it cannot be substituted for tissue analysis (**Table 3**).

**Table 3:** The results of gene mutations for brain parenchyma metastases.

NO.	Driver mutations of primary tumour	Interval time (month)	Tumour tissues	Abundance(tissues)	Plasma ctDNA	Abundance (plasma)	CSF ctDNA
1	unknown	12			19del	0.3%	(-)
2	19del	132			(-)		(-)
3	19del	0	19del	31.4%	POLE	0.6%	(-)
4	KRAS	0			KRAS	17.3%	(-)
5	L858R/A871E/MET	0	L858R/A871E/MET	39.5%/38.8%/19.2%	L858R/A871E/MET	0.1%/0.2%/0.1%	Unperformed
6	ROS1	18			DNMT3A	0.5%	Unperformed
7	19del	23			19del/T790M	0.1%/0.2%	(-)
8	19del	0	19del	25.3%	19del	2.3%	(-)
9	ALK	0	ALK	24.2%	(-)		(-)

CSF, cerebrospinal fluid circulating tumour; ctDNA, circulating tumour DNA; NSCLC, non-small cell lung cancer.

## Discussion

In our study, the median interval from the NSCLC diagnosis to BM was 18.0 (0.0–70.0) months. BM incidence can increase to 80% in some particular groups, such as patients with anaplastic lymphoma kinase (*ALK*) positive NSCLC patients (8). This is particularly important for Asians, whose prevalence of *EGFR* mutations has been reported to be 63%, much higher than other populations (1, 9). In our study, the most frequently detected driver mutations in BM were *EGFR* mutations (23/37, 62.2%), including L858R (13/37, 35.1%) and 19del (10/37, 27.1%), respectively, which is consistent with the results of previous studies (1, 10).

Genetic mutation profiles are fundamental to precision medicine in patients with tumours (11). With the development of liquid biopsy and sequencing methods, plasma and CSF ctDNA detected by NGS play increasingly important roles in guiding the management of NSCLC-BM. Previous studies have shown that CSF ctDNA can reflect BM’s molecular characteristics and heterogeneity, including BPM (12, 13). In our study, the CSF ctDNA positivity rate was high in patients with LM but low in patients with BPM (all tested patients were negative). A previous study indicated that the detection rate of mutations in the CSF was lower in patients with BPM, which might be because tumours are located farther away from the cerebral ventricle, where CSF is generated. Cancer cells may access the leptomeningeal space through four main points of entry: arterial circulation through the choroid plexus, venous circulation through Bateson’s plexus, direct invasion along the spinal and cranial nerves, and invasion from parenchymal disease through the glia limitans (14).

The process of tumour cell metastasising to the brain involves a series of steps, including detachment from the primary site, invasion of surrounding tissues and blood vessels, blood transmission, crossing of the blood-brain barrier (BBB), and brain clonal growth (14). In our study, the absence of CSF ctDNA in patients with BPM may be because the tumour cells had not yet crossed the BBB. To some extent, CSF ctDNA combined with imaging manifestations may assist clinicians in determining whether patients with BPM and/or LM require intrathecal chemotherapy. If conditions permit, we suggest that patients with BPM should be simultaneously tested for CSF ctDNA, plasma ctDNA, and tumour tissues for comprehensive evaluation and treatment. Further studies of other sensitive biomarkers and advanced testing methods for BMP are required.

The CSF ctDNA levels may reflect the molecular characteristics and heterogeneity of patients with NSCLC-LM and complement the LM diagnosis (15). A previous study showed that the mutation status of NSCLC-LM patients was concordant with the primary tumour and is in approximately 90% of cases (16). In our study, the mutation profile of patients with NSCLC-LM patients in CSF showed high concordance with the primary tumours (90.5%), consistent with the result of a previous study. Many other mutations have also been detected in CSF, reflecting tumour heterogeneity. This result suggested that CSF ctDNA is a reliable biomarker that may guide the management of NSCLC-LM. In particular, plasma ctDNA is the most extensively studied and widely used method for genotyping if tumour tissue is not available (17), which mostly reflects the primary tumours and extracranial disease (13).

As the application time of TKI drugs is extended, the tumour cells develop new mutations, or the non-dominant mutations become dominant mutations, leading to drug resistance (18). In our study, the driver mutations in patients with LM included *EGFR* L858R (12, 42.9%), 19del (6, 21.4%), *ALK* (2, 7.1%), and *ROS1* (2, 7.1%), which are the major drug targets in the clinic. Third-generation TKI drugs with high BBB permeability are recommended for patients with LM even if there is no T790M mutation. In the present study, the most common drug-resistant mutations were *EGFR* T790M (3/28, 10.7%) and *MET* amplification (3/28,10.7%). A previous study showed that the *EGFR* T790M mutation is the main resistance mechanism generated after applying first- and second-generation *EGFR*-TKI drugs, which could be easily detected in the plasma (19). In our study, three patients were *EGFR* T790M mutation positive, of which two cases were detected in the plasma samples and one case in the CSF sample. All three patients were administered first-generation TKIs. To date, osimertinib has been approved for patients harbouring the *EGFR* T790M mutation, which is suitable for LM treatment (20). MET amplification is another mechanism underlying acquired TKI drug resistance (21). Acquired *MET* amplification has been identified in 5%–20% of NSCLC patients with sensitive *EGFR* mutations, who develop resistance to first-, second-, and third-generation *EGFR*-TKIs (22). In our study, three patients treated with third-generation TKIs showed *MET* amplification, all of which were detected in the CSF. The combination of an *EGFR*-TKI and *MET*-TKI remains effective for NSCLC patients with both *EGFR* mutations and *MET* amplification after progression to a prior *EGFR*-TKI, especially for patients with higher levels of *MET* amplification (23).

*EGFR* G719C, G719S, L861Q, S768I, and C797S were also detected in our study. Previous studies show that the incidence of uncommon *EGFR* mutations accounts for approximately 20% of *EGFR*-mutated NSCLC patients (24). The G719X and L861Q are the main uncommon mutations and are associated with favourable efficacy of *EGFR*-TKIs (25), whereas the S768I mutation is in exon 20 and is associated with a lack of sensitivity to *EGFR*-TKIs (26). The combination of C797S with T790M mutation is a reason for osimertinib resistance (27). A previous study indicated that the prognosis of patients with uncommon mutations was significantly inferior to that of patients with common mutations (including L858R and 19del mutations) (26). Afatinib, a second-generation *EGFR*-TKI, appears to be able to penetrate the CNS at a sufficient concentration to have a clinical effect on CNS metastases and might be the optimal *EGFR*-TKI against these uncommon *EGFR* mutations (28). In addition, most patients with LM not harbouring resistance genes also showed disease progression, suggesting the existence of other resistance mechanisms or the limitations of current detection methods. In our study, many patients with LM harboured *TP53* (17/28, 60.7%) mutations. *TP53* is a tumour suppressor gene and mutations in this gene predict poor prognosis. To date, *TP53* mutations are undruggable (29).

*KRAS* mutations have been considered a key driver of lung cancer, in which *KRAS* p.G12C accounts for 45% to 50% of *KRAS* mutations (30). In our study, three patients with NSCLC-LM harboured *KRAS* mutations, all of which were p.G12V mutations (3/28, 10.7%), two of which were detected in the CSF and one in the plasma. Despite recent drug developments with some drugs targeting *KRAS* p.G12C mutation, most *KRAS* oncoproteins remain undruggable (31).

Most patients with advanced NSCLC (68.1%) had BPM comorbidities. However, only 32.4% of the patients were simultaneously diagnosed with LM and advanced NSCLC. Most patients with NSCLC are diagnosed with LM onset during the treatment course, which is considered a later event than BPM in advanced NSCLC. In addition, compared to BPM, LM patients are prone to multiple metastases (≥ 2 metastatic sites) and have shorter survival times (1).

Cancers are prone to complications. Melanoma, NSCLC, small-cell lung cancer, and breast cancer are prone to both BPM and LM. Renal cancers often metastasise to the brain parenchyma. In contrast, lymphomas and leukaemia often cause LM. Patients with small-cell lung cancer, adenocarcinoma of the lung, and non–small cell lung cancer have an incidence of brain metastases at diagnosis of >10%. Only 0.4%, 1.5%, and 0.7% of patients with breast cancer, renal cancer, and melanoma, respectively, had brain metastases at diagnosis (32).

The mechanisms underlying these phenomena are unknown, but they may be clinical manifestations of genetic aberrations in different tumours. NSCLC patients with *EGFR* L858R are more likely to develop LM than those with *EGFR* 19del (1). In a BPM study, Takano et al. found that *EGFR* L858R mutation metastases were more likely to occur in the parenchyma (caudate, cerebellum, and temporal lobes) than those with 19del and were located closer to the surface of the brain than those with 19del or wild-type *EGFR* (33).

However, the limited sample size of nine patients with BMP did not allow us to thoroughly compare the characteristics of BPM and LM. Our study showed that 55.6% (5/9) of patients with BMP harboured *EGFR* 19del mutation, whereas only 11.1% (1/9) of them harboured the L858R mutation. In contrast, 50.0% (14/28) of patients with LM harboured *EGFR* L858R, while 25.0% (7/28) of them harboured *EGFR* 19del mutation. Similar results were reported by Li et al. that NSCLC patients with *EGFR* 19del were more likely to develop BMP than patients with the *EGFR* L858R mutation (1).

We also found that there was a sex difference in the extracranial disease of NSCLS-LM patients. Patients with positive driver mutations in plasma ctDNA were mostly male (M:F = 9:4) and were more likely to be at a progressive stage (8 out of 13, 61.5%), suggesting that male patients had a higher tumour burden (**Tables 1**, **2**, **Figure 3A**, **Supplementary Figure 1**). Accumulating evidence shows that the incidence of lung cancer is higher in males than in females; furthermore, male patients had a poorer prognosis than female patients (34). NSCLC female patients are usually non-smokers, and *EGFR* mutation positive. *EGFR* incidence is especially high in Asian NSCLC patients (35). The better outcome for female patients might be because they could be treated with *EGFR* TKIs, which was consistent with clinical practice.

In addition, in LM patients who were ctDNA positive in the plasma, the extracranial disease was more likely to be in a progressive stage compared to negative patients, 61.5% (8/13) versus 13.3% (2/15) (**Tables 1**, **2**, **Figure 3A**), suggesting that ctDNA in the plasma is a prognostic factor. This may be because a higher tumour burden causes higher ctDNA levels in the plasma. Our results suggest that plasma ctDNA is a prognostic factor for patients with NSCLC-LM (10, 36).

To the best of our knowledge, few studies have used NGS to compare cancer-related genetic profiles of NSCLC-LM and NSCLC-BPM patients (1). Li et al. compared the characteristics of patients with NSCLC-LM and NSCLC-BPM in Sichuan province, located in southeastern China, including *EGFR* mutations, onset time of BPM or LM, proportion of multiple metastases, and survival. They found significant differences in lesion location and *EGFR* mutation subtypes between patients with NSCLC-LM and those with NSCLC-BPM. Our study included patients from the Hebei Province, located in the northern part of China. These two studies represent the characteristics of the Chinese population. Further studies should be conducted in other ethnicities, such as Caucasians and Africans, to determine whether this is a universal phenomenon.

The present study has limitations. Our study was a single-centre retrospective study with a relatively small sample size and case selection was based on the NGS of CSF and paired plasma, which had inevitable bias in case selection. Therefore, future multi-centre, prospective large sample-sized studies are needed to validate our findings.

## Conclusions

Our study indicated that the main driver mutations of NSCLC-LM remained highly consistent with those of primary tumours, along with other unique genetic profiles. CSF ctDNA detected by NGS may reflect the molecular characteristics and heterogeneity of NSCLC-LM. Timely screening of NSCLC patients for CSF ctDNA, especially for patients with a mutation in plasma ctDNA, may facilitate early detection of LM. Patients with *EGFR* 19del might be at higher risk of suffering from BPM.

## Data availability statement

The raw sequence data reported in this paper have been deposited in the Genome Sequence Archive (37) in National Genomics Data Center (38), China National Center for Bioinformation / Beijing Institute of Genomics, Chinese Academy of Sciences (GSA-Human: HRA006432) that are publicly accessible at https://ngdc.cncb.ac.cn/gsa-human.

## Ethics statement

The studies involving humans were approved by The Institutional Review Board and Ethics Committee of the Fourth Hospital of Hebei Medical University. The studies were conducted in accordance with the local legislation and institutional requirements. The participants provided their written informed consent to participate in this study.

## Author contributions

XL: Data curation, Investigation, Writing – original draft. FM: Funding acquisition, Methodology, Project administration, Supervision, Writing – original draft, Writing – review & editing. MF: Data curation, Formal Analysis, Funding acquisition, Supervision, Writing – original draft, Writing – review & editing. YJ: Data curation, Formal Analysis, Investigation, Methodology, Writing – original draft. YZ: Data curation, Investigation, Software, Writing – original draft. CXL: Data curation, Formal Analysis, Methodology, Validation, Writing – original draft. PT: Data curation, Formal Analysis, Investigation, Methodology, Writing – original draft. CFL: Data curation, Formal Analysis, Investigation, Software, Validation, Writing – original draft. GL: Conceptualization, Data curation, Formal Analysis, Funding acquisition, Investigation, Methodology, Project administration, Resources, Supervision, Validation, Writing – original draft, Writing – review & editing.
