# Supplementary material for: Cerebrospinal fluid ctDNA testing shows an advantage over plasma ctDNA testing in advanced non-small cell lung cancer patients with brain metastases
Source: Front Oncol. 2024 Jan 10;13:1322635. doi: 10.3389/fonc.2023.1322635 (PMC10806520; doi:10.3389/fonc.2023.1322635)
Supplement: Supplementary file 1 [file DataSheet_1.docx]

Supplementary Material

**Supplementary Table 1:** The 425 cancer related genes detected by NGS in CSF or plasma samples

| ABCB1(MDR1) | CKD12 | ERCC2 | IDH1 | MSH6 | PPARD | SMO |
| --- | --- | --- | --- | --- | --- | --- |
| ABCC2(MRP2) | CKD4 | ERCC3 | IDH2 | MTHFR | PPP2R1A | SOCS1 |
| ADH1B | CDK6 | ERCC4 | IFNA6 | MTOR | PRDM1 | SOS1 |
| AIP | CDK8 | ERCC5 | IFNB1 | MUTYH | PREX2 | SOX2 |
| AKT1 | CDKN18 | ESR1 | IFNE | MYC | PRF1 | SPOP |
| AKT2 | CDKN1B | ETV1 | IFNG | MYCL | PRKACA | SPRED1 |
| AKT3 | CDKN1C | ETV4 | IFNGR1 | MYCN | PRKAR1A | SPRY4 |
| ALDH2 | CDKN2A | ETV5 | IFNGR2 | MYD88 | PRKCI | SRC |
| ALK | CDKN2B | ETV6 | IGF1R | MYH9 | PRKDC | SRSF2 |
| AMER1 | CDKN2C | EWSR1 | IGR2 | NAT1 | PRSS1 | SRY |
| APC | CEBPA | EXT1 | IKBKE | NBN | PRSS3 | STAG2 |
| AR | CEP57 | EXT2 | IKZF1 | NCOR1 | PTCH1 | STAT3 |
| ARAF | CHD4 | EZH2 | IL7R | NF1 | PTEN | STK11 |
| ARID1A | CHD8 | EZR | INPP4B | NF2 | PTK2 | STMN1 |
| ARID1B | CHEK1 | FANCA | IRF2 | NFE2L2 | PTPN11 | SUFU |
| ARID2 | CHEK2 | FANCC | JAK1 | NFKBIA | PTPN13 | TACC3 |
| ARID5B | CREBBP | FANCD2 | JAK2 | NKX2-1 | QKI | TAP1 |
| ASCL4 | CRKL | FANCE | JAK3 | NOTCH1 | RAC1 | TAP2 |
| ASXL1 | CSF1R | FANCF | JARID2 | NOTCH2 | RAC3 | TEK |
| ATF1 | CTCF | FANCG | JUN | NOTCH3 | RAD50 | TEKT4 |
| ATIC | CTLA4 | FANCI | KDM5A | NPM1 | RAD51 | TERC |
| ATM | CTNNB1 | FANCL | KDR(EGFR2) | NQO1 | RAD51B | TERT |
| ATR | CUL3 | FANCM | KEP1 | NRAS | RAD51C | TET2 |
| ATRX | CUX1 | FAT1 | KIF1B | NRG1 | RAD51D | TGFBR2 |
| AURKA | CXCR4 | FBXW7 | KIT | NSD1 | RAD54L | THADA |
| ARUKB | CYLD | FGF19 | KITLG | NTRK1 | RAF1 | TMEM127 |
| AXIN2 | CYP19A1 | FGFR1 | KLLN | NTRK2 | RARA | TMPRSS2 |
| AXL | CYP2A13 | FGFR2 | KMT2A(MLL) | NTRK3 | RARG | TNFAIP3 |
| B2M | CYP2A6C | FGFR3 | KMT2B | NUTM1 | RASGEF1A | TNFRSF11A |
| BAD | CYP2A7 | FGFR4 | KMT2C | PAK3 | RB1 | TNFRSF14 |
| BAI3 | CYP2B6*6 | FH | KMT2D(MLL2) | PALB2 | RECQL4 | TNFRSF19 |
| BAK1 | CYP2C19*2 | FLCN | KRAS | PALLD | RELN | TNFSF11 |
| BAP1 | CYP2C9*3 | FLT1(VEGFR1) | LHCGR | PARK2 | RET | TOP1 |
| BARD1 | CYP2D6 | FLT3 | LMO1 | PARP1 | RHOA | TOP2A |
| BAX | CYP3A4*4 | FLT4 | LRP1B | PARP2 | RICTOR | TP53 |
| BCL2 | CYP3A5 | FOXA1 | LYN | PAX5 | RNF43 | TP63 |
| BCL2L11(BIM) | CYSLTR2 | FOXL2 | LZTR1 | PBRM1 | ROS1 | TPMT |
| BCR | DAXX | FOXP1 | MAP2K1(MEK1) | PDCD1(PD1) | RPTOR | TSC1 |
| BIRC3 | DDR2 | FRG1 | MAP2K2(MEK2) | PDCD1LG2(PD-L2) | RRM1 | TSC2 |
| BLM | DENND1A | GATA1 | MAP2K4 | PED11A | RUNX1 | TSHR |
| BMPR1A | DHFR | GATA2 | MAP3K1 | PDGFRA | RUNX1T1 | TTF1 |
| BRAF | DICER1 | GATA3 | MAP3K4 | PDGFRB | SBDS | TUBB3 |
| BRCA1 | DLL3 | GATA4 | MAX | PKD1 | SDC4 | TYMS |
| BRCA2 | DNMT3A | GATA6 | MCL1 | PGR | SDHA | U2AF1 |
| BRD4 | DOT1L | GNA11 | MDM2 | PHOX2B | SDHB | UGT1A1 |
| BRIP1 | DPYD | GNAQ | MDM4 | PIK3C3 | SDHC | VAMP2 |
| BTG2 | DTL(CDT2) | GNAS | MECOM | PIK3CA | SDHD | VEGFA |
| BTK | DUSP2 | GRIN2A | MED12 | PIK3CD | SEPT9 | VHL |
| BUB1B | EGFR | GRM3 | MEF2B | PIK3R1 | SETBP1 | WAS |
| c11orf30 | EIF1AX | GRM8 | MEN1 | PIK3R2 | SETD2 | WISP3 |
| CASP8 | EP300 | GSTM1 | MET | PKHD1 | SF3B1 | WRN |
| CBL | EPAS1 | GSTM4 | MGMT | PLAG1 | SGK1 | WT1 |
| CBLB | EPCAM | GSTP1 | MITF | PLCB4 | SKP2 | XPA |
| CCND1 | EPHA2 | GSTT1 | MLH1 | PLK1 | SLC34A2 | XPC |
| CCNE1 | EPHA3 | HDAC2 | MLH3 | PMS1 | SLC3A2 | XRCC1 |
| CD274(PD-L1) | EPHA5 | HDAC9 | MLLT1 | PMS2 | SMAD2 | XRCC2 |
| CD74 | ERBB2(HER2) | HGF | MLLT3 | POLD1 | SMAD3 | YAP1 |
| CDA | ERBB2IP | HLA-A | MLLT4 | POLD3 | SMAD4 | ZNF217 |
| CDC73 | ERBB3 | HNF1A | MPL | POLE | SMAD7 | ZNF703 |
| CDH1 | ERBB4 | HNF1B | MRE11A | POLH | SMARCA4 |  |
| CKD10 | ERCC1 | HRAS | MSH2 | POT1 | SMARCB1 |  |


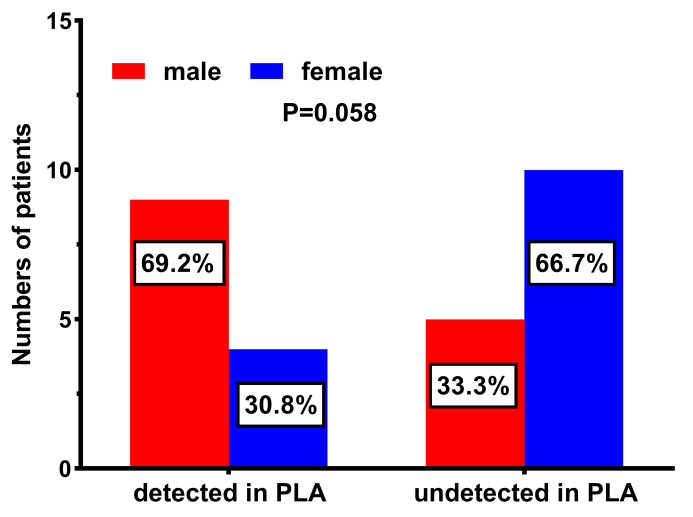


**Supplementary Figure 1:** Distribution of sex in NSCLC-LM patients who were ctDNA positive or negative in plasma samples.
